# Comparative evaluation of plan robustness in proton and photon radiotherapy in deep inspiration breath-hold for mediastinal lymphomas throughout the treatment course

**DOI:** 10.1016/j.phro.2026.101111

**Published:** 2026-10-22

**Authors:** Filip Hörberger, Marika Enmark, Karin M. Andersson, Rebecka Ericsson Szecsenyi, Gracinda Johansson, Daniel Molin, Kenneth Wikström, Ingrid Kristensen, Anna Flejmer, Anneli Edvardsson

**Affiliations:** a Medical Radiation Physics, Department of Clinical Sciences Lund, Lund University, Lund, Sweden; b Radiation Physics, Department of Hematology, Oncology and Radiation Physics, Skåne University Hospital, Lund, Sweden; c Department of Medical Physics, The Skandion Clinic, Uppsala, Sweden; d Department of Medical Physics, Uppsala University Hospital, Uppsala, Sweden; e Department of Immunology, Genetics and Pathology: Cancer Immunotherapy, Uppsala University, Uppsala, Sweden; f Department of Immunology, Genetics and Pathology, Uppsala University, Uppsala, Sweden; g Department of Clinical Sciences, Oncology, Lund University, Lund, Sweden; h Department of Oncology, Uppsala University Hospital, Uppsala, Sweden

**Keywords:** Proton therapy, VMAT, Mediastinal lymphomas, Deep inspiration breath-hold, Robust optimization, Repeated verification CTs

## Abstract

**Background and purpose:**

Proton therapy (PT) is sensitive to anatomical changes and setup uncertainties, which can compromise dose robustness in the treatment of mediastinal lymphoma, an issue less pronounced with photon-based radiotherapy. Variations in target position and size can potentially influence plan robustness. This study evaluated the robustness of pencil beam scanning (PBS) PT versus volumetric modulated arc therapy (VMAT) photon treatment plans across sub-target volumes and organs of interest (OOI) in deep inspiration breath-hold (DIBH) throughout treatment.

**Material and methods:**

Twenty-eight patients with upper mediastinal lymphoma treated with PBS-PT in DIBH were included. Clinically delivered PBS-PT plans were retrospectively compared with VMAT plans optimized per institutional standard-of-care. All plans were recalculated on weekly verification computed tomography images (vCTs) to assess target coverage and OOI doses. The internal target volumes (ITV) were subdivided into mediastinum, fossa supraclavicularis, neck, axilla and, along with OOIs, transferred from planning CTs to the vCTs.

**Results:**

In the nominal plans, PBS-PT generally achieved lower OOI doses than VMAT, an advantage maintained on the vCTs. Both modalities demonstrated robust target coverage and OOI sparing. ITV_Total_ coverage fell below the set criterion (D_98%_ > 95%) on at least one vCT for six patients with PBS-PT, but only one patient for VMAT. Pronounced deviations (D_98%_ < 90%) occurred for two PBS-PT cases. Sub-target robustness was generally good.

**Conclusions:**

Both PBS-PT and VMAT were robust for mediastinal lymphoma treatment in DIBH. In a proton-eligible DIBH cohort, PBS-PT provided superior OOI sparing, while VMAT demonstrated greater consistency in target coverage.

## Highlights

• Longitudinal comparison of plan robustness between proton and photon plans.
• Clinically delivered proton therapy plans in breath-hold for mediastinal lymphoma.
• Target coverage evaluation of subdivided target volumes, e.g. mediastinum and neck.
• Protons reduced median breast, lung, and heart doses by 3.6, 2.6, and 2.0 Gy (RBE).
• Target underdosage on verification CTs occurred in 1/28 photon and 6/28 proton plans.

## Graphical abstract

**Figure:**
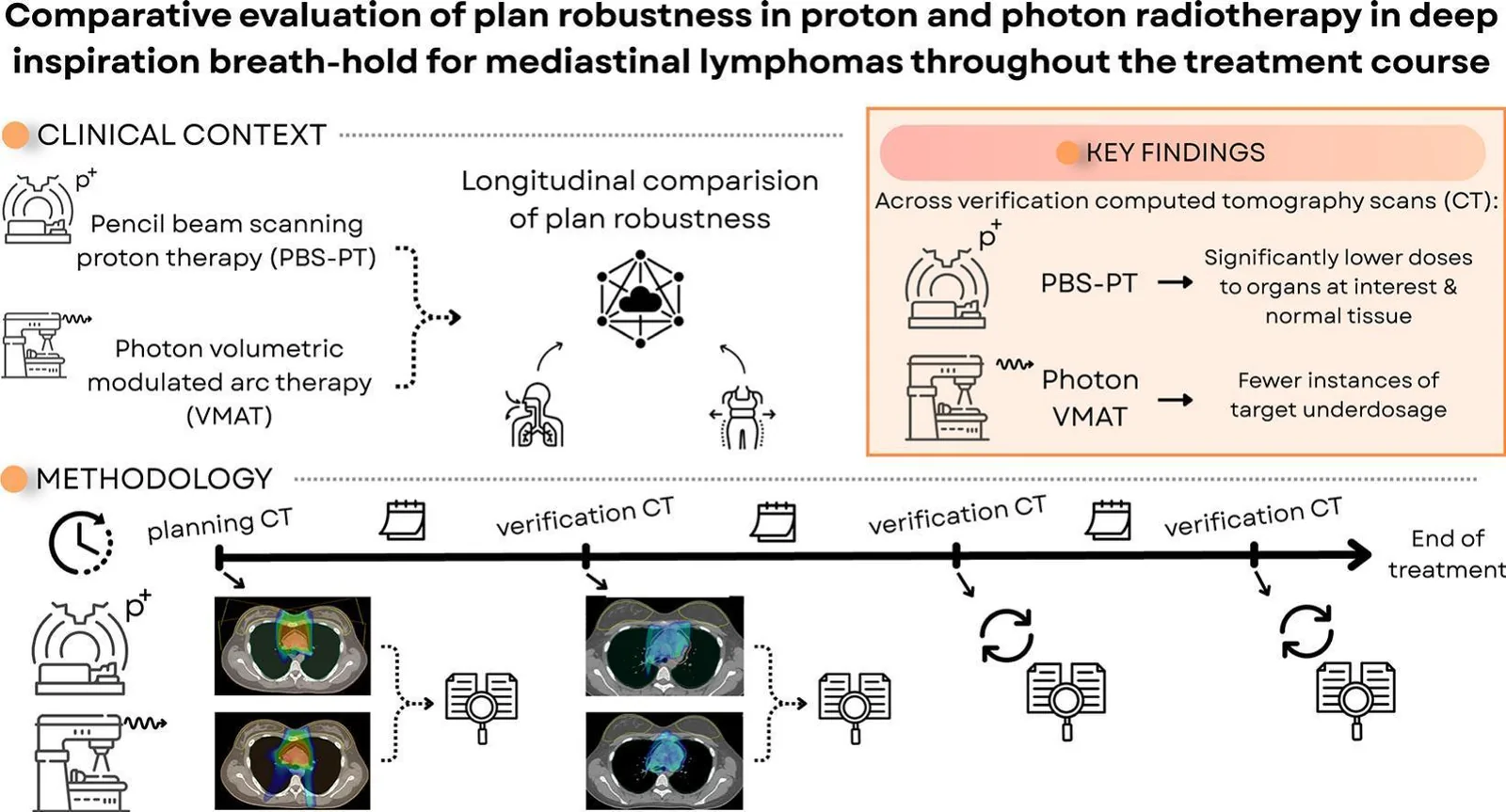

## 1 Introduction

Over the past few decades, the management of mediastinal lymphomas has improved substantially, largely due to the integration of chemotherapy and radiotherapy (RT), resulting in cure rates exceeding 80–90% [1], [2]. As more patients become long-term survivors, the focus has increasingly shifted toward minimizing the late effects of treatment, particularly radiation-induced side effects such as secondary malignancies and cardiovascular disease [3], [4], [5]. This has driven efforts to optimize treatment approaches that maintain high disease control while reducing both prescribed dose and irradiated volume [6], [7], [8].

Advanced photon-based techniques, such as volumetric modulated arc therapy (VMAT) have been developed to enable more conformal dose distributions. However, a key limitation of VMAT remains the tendency to deliver a wider low-dose bath, resulting in larger volumes of normal tissue (NT) and organs of interest (OOI) being irradiated. Proton therapy (PT), offering beam characteristics with finite range and steep distal dose fall-off, reduces the low-dose bath compared to photon-based techniques [9], [10]. These characteristics are advantageous in the treatment of mediastinal lymphomas, where critical organs such as the heart and lungs are located in close proximity to the target volume [2], [11], [12], [13].

However, the use of PT in the thoracic region presents challenges due to respiratory motion, which can severely degrade the planned dose distribution through interplay effects and/or range uncertainties caused by anatomical variations [14], [15], [16], [17]. To mitigate these uncertainties, motion management strategies, e.g., deep inspiration breath-hold (DIBH), are commonly employed. DIBH can create an almost static anatomical setup as well as displacing the target volume into a more favorable position with respect to the OOIs, thereby reducing OOI doses [7], [8], [18]. Nevertheless, good inter-breath-hold reproducibility is critical for maintaining adequate target coverage and effective OOI sparing, whereas insufficient breath-hold reproducibility can require replanning [19].

Although several studies have demonstrated OOI dose sparing advantages at the nominal plan level for PT compared to photon RT both in free breathing and DIBH for mediastinal lymphoma [7], [18], [20], [21], comprehensive assessments of treatment robustness throughout the treatment course between the two modalities are still lacking. In addition, lymphomas occur in a wide range of anatomical locations and sizes, therefore, this study aimed to compare plan robustness to sub-target volumes and OOIs between pencil beam scanning (PBS) PT delivered in DIBH with retrospectively optimized VMAT treatment plan (TPs), by recalculation on weekly verification computed tomography (vCTs) acquired throughout the PBS-PT treatment course.

## 2 Material and methods

### 2.1 Patients

All patients included in this study (*n* = 28) were diagnosed with upper mediastinal lymphoma, with or without supraclavicular fossa, neck or axillary involvement, and underwent PBS-PT in DIBH between February 2018 and May 2023. This represents all patients treated with PBS-PT in DIBH from Skåne University Hospital and Uppsala University Hospital up to May 2023. Detailed patient characteristics are presented in Table 1. Eleven of the patients were treated within the PRO-Hodgkin trial [6]. Treatment was administered at the national proton therapy facility in Sweden, the Skandion Clinic, with preparatory procedures conducted at the University Hospitals [22].

**Table 1:** Patient and treatment characteristics. Abbreviations include ITV - Internal target volume, Med - Mediastinum and FSC - Fossa supraclavicularis.

Age (years)	Median (range): 27 (17–64)
Gender:	No. of patients:
Female	16
	12

Diagnosis:	No. of patients:
Classical Hodgkin lymphoma	22
Primary mediastinal B-cell lymphoma	6

Stage:	No. of patients:
I-A, I-B	4, 1
II-A, II-B, II-E	16, 2, 1
IV-A	2
Bulky	2

Disease location:	No. of patients:
Med	13
Med + FSC	7
Med + axilla	1
Med + FSC + neck	5
Med + FSC + neck + axilla	2

ITV volume:	Median (range) [cm^3^]:
ITV_Total_	239 (73–1109)
ITV_Med_	142 (7–767)
ITV_FSC_	90 (3–314)
ITV_Neck_	36 (6–85)
ITV_Axilla_	158 (13–270)

Dose [Gy_(RBE)_] x fractions:	No. of patients:
2.00 × 10	4
1.80 × 11	1
2.00 × 15	5
1.75 × 17	16
1.80 × 20	1
2.00 × 20	1

No. of verification CTs:	No of patients:
2	7
3	13
4	6
5	2

The usage of radiotherapy data for retrospective research was approved by the Regional Ethical Review Board in Lund and Uppsala, Sweden (No.2013/742 and No. 2019-00010).

### 2.2 CT imaging and deep inspiration breath-hold

Planning CT (pCT) images in DIBH were acquired with a Siemens SOMATOM Definition AS (Siemens Healthineers, Erlangen, Germany) or a Philips Brilliance Big Bore 16-slice (Philips Medical Systems, Best, Netherlands) CT scanner (slice thickness of 2–3 mm and in-plane pixel size of 1 × 1 mm^2^). All patients were positioned head-first supine, 19 patients with treated arms-up and 9 with arms-down in combination with a thermoplastic mask. For DIBH monitoring, the vertical displacement of the chest wall was tracked using the surface scanning system Sentinel™ (C-RAD Positioning AB, Uppsala, Sweden). Patients were guided by audio cues and real-time visual feedback via video goggles, with identical monitoring applied during treatment and vCT acquisition. The clinical workflow has previously been described in more detail elsewhere [18], [19].

Prior to the first treatment fraction, a CT scan in DIBH was repeated to verify the patients' DIBH-reproducibility and assess other potential anatomical changes. Thereafter, repeated DIBH CT-scans were generally acquired once per week throughout the PT treatment course, or when deemed necessary. All CT scans acquired after the initial pCT, are hereafter referred to as vCTs. For each patient, between 2 and 5 vCTs were collected, resulting in a total of 87 vCTs across the patient cohort (Table 1).

### 2.3 Target definition and contouring

The gross tumor volume (GTV) was defined as the residual tumor identified on fluorodeoxyglucose positron emission tomography. The clinical target volume (CTV) encompassed the initial extent of disease, including the GTV. To account for breath-hold variability during DIBH, an internal target volume (ITV_Total_) was generated based on repeated DIBH-CT (one pCT and two low-dose CT acquired at the same session), as previously described elsewhere [18], [19]. For this study, the ITV was retrospectively subdivided into smaller sub-ITVs based on anatomical location, categorized as Mediastinum (ITV_Med_), Neck (ITV_Neck_), Fossa supraclavicularis (ITV_FSC_) and Axilla (ITV_Axill_),^1^ see Fig. 1. The planning target volume (PTV) was generated by expanding the ITV with an additional 5 or 7 mm isotropic patient specific margin. OOIs included the heart, lungs, esophagus, and breasts (female patients). All structures were transferred from the pCT to the vCTs with deformable image registration, based on the Demons algorithm in Eclipse*™* treatment planning system (TPS) (Varian Medical Systems, Milpitas, CA). All transferred structures were reviewed by an oncologist, with no manual editing required.

**Fig. 1:**
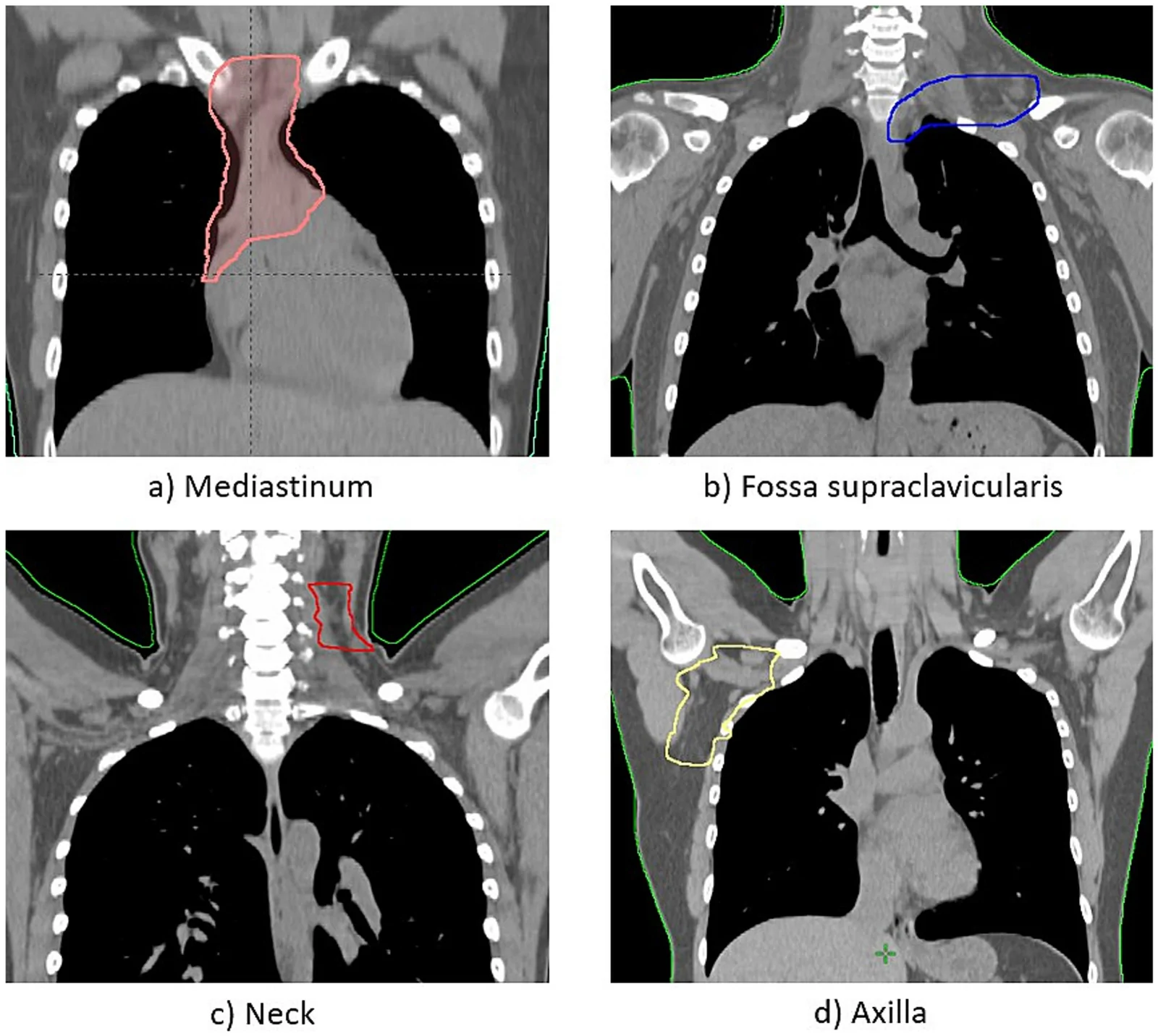
Representative illustrations of internal target volumes, including (a) Mediastinum, (b) Fossa supraclavicularis, (c) Neck and (d) Axilla, for patients enrolled in the study.

### 2.4 Treatment planning

The PBS-PT TPs were robustly optimized (RO) on the ITV_**Total**_ using the Nonlinear Universal Proton Optimizer (V13.7.15–15.6.03) and calculated with the Proton Convolution Superposition algorithm (V13.7.15–15.6.04) in Eclipse™ TPS. A relative biological effectiveness (RBE) of 1.1 was applied [23], [24]. Typically, two to three anterior oblique beams (gantry angles 335°–25°) were used, with selection based on target location, OOIs, and density heterogeneities along the beam path. A range shifter (water-equivalent thickness of 3.5 cm) was generally used to increase the spot size [25], reducing interplay effects [26]. Spot-spacing varied between 0.3 and 0.7 cm. Single field uniform dose (SFUD) optimization was used for 20 patients and multifield optimization (MFO) for eight patients. RO with set-up uncertainties of 5 or 7 mm (corresponding to the isotropic, patient specific, ITV_Total_-PTV margin) and a range-uncertainty of 4.5% [27] were used. All plans underwent robust evaluation under 5 or 7 mm and 4.5% perturbation conditions to ensure adequate target coverage and OOI sparing. At least 10/12 uncertainty scenarios had to meet the institutional criteria (*D*_98%,ITV_ ≥ 95%), with the goal of satisfying it in as many scenarios as possible. OOIs were robustly evaluated based on national guidelines [6].

The comparative photon TPs were created as per institutional standard-of-care. All VMAT TPs were optimized on the PTV, using as few arcs as possible (generally one arc, maximum of two), to reduce treatment time and thereby the required number of breath-holds. Moreover, two different TPSs were used, corresponding to those in clinical use at the participating institutions. 21 plans were optimized with the Photon optimizer algorithm (V18.0.1) and the AAA dose calculation algorithm (V15.6.05) in the Eclipse™ TPS. The aperture shape controller setting was set to “Very High” (for one patient moderate was used) to limit the complexity of multi-leaf collimator movement. The other seven VMAT-plans were optimized and calculated in Monaco (V6.2.2.0 Elekta Solutions AB, Stockholm, Sweden), using Monte Carlo with a 1% statistical uncertainty. Due to partial PTV extension into the lung, the planning objective for all photon TPs was to achieve *D*_100%,PTV_ ≥ 93%, while maintaining ITV coverage as defined above.

All PBS-PT and VMAT TPs were determined to be robust and clinically acceptable at the planning stage. Representative beam angles and dose distributions are illustrated in Fig. 3a for both modalities.

### 2.5 Verification CT evaluation and statistical analysis

All TPs were recalculated on the vCTs, with the isocenter transferred using rigid registration based on bony anatomy. For each RT-modality, ITV coverage (*D*_98%,ITV_) and OOI doses were extracted from the vCTs and compared with the corresponding values from the nominal pCTs. The following DVH-parameters were analyzed: *D*_98%,ITV_, $\bar{D}$_Heart_, $\bar{D}$_LungTotal_, $\bar{D}$_L/R Breast_, *D*_2%,Esophagus_, *D*_*0.5cm*_ *3*_*,Body*_, *V*_*30%,*Body_, V_15Gy(RBE),Heart_, *V*_5Gy(RBE),L/R Breast_, *V*_5Gy(RBE),LungTotal_ and *V*_20Gy(RBE),LungTotal_ (where $\bar{D}$ is the mean dose).

A mixed-effects model was used to assess statistical significance (Anaconda, Python 3.13.9) of DVH-parameter differences between RT techniques (PT vs VMAT) for vCTs, as well as between pCTs and vCTs within each technique. For comparisons between the two modalities on the nominal stage (pCTs), a Wilcoxon test was applied because the test included no repeated measurements (i.e., multiple vCTs). Given the multiple DVH-endpoint tests (*n* = 15), a Bonferroni-corrected significance threshold was used (*p* < 0.003), corresponding to an overall α = 0.05.

## 3 Results

Generally, PBS-PT significantly (*p* < 0.003) reduced the dose to OOIs, and NT compared with VMAT, and these benefits were maintained in recalculations on vCTs (Table 2). Notably on the vCTs, the median low-dose region (V_30%,Body_) decreased by 1800 cm^3^ with PT compared to VMAT. Additionally, the median $\bar{D}$ reduction for the breasts, lungs and heart was 3.6 Gy(RBE), 2.6 Gy(RBE) and 2.0 Gy(RBE), respectively, with PBS-PT compared to VMAT. However, a significantly higher V_20Gy(RBE),LungTotal_ was observed with PBS-PT, by approximately 1.8 percentage points (*p* < 0.003). Although target coverage was generally comparable between techniques, ITV_Total_ and ITV_Med_ D_98%_ were significantly lower with PBS-PT across vCTs, by 1.4 and 1.2 percentage points, respectively (*p* < 0.003).

**Table 2:** Comparison of DVH parameters between modalities at planning CTs (nominal) and verification CTs, presented as median value and range. A Wilcoxon test (*p* < 0.003) was used to assess statistically significant differences at the nominal stage between the modalities, whereas a mixed-effect model (*p* < 0.003) was used for comparison at the verification CTs imaging stage. No statistical comparison was performed on the neck target (ITV_Neck_) and axilla (ITV_Axill_) due to the limited number of data points.

	Planning CT			Verification CT		
DVH parameter	PBS-PT	VMAT	*P*-Value	PBS-PT	VMAT	P-Value
ITV_Total_ D_98%_ [%] (*n* = 28)	97.8 (96.9–99.9)	98.2 (95.4–100.4)	0.130	97.4 (77.9–99.5)	98.1 (94.5–101.2)	< 0.003
ITV_Med_ D_98%_ [%] (n = 28)	97.7 (96.8–100.2)	98.3 (94.5–100.0)	0.210	97.4 (75.9–100)	98.1 (94.6–101.1)	< 0.003
ITV_FSC_ D_98%_ [%] (*n* = 14)	98.2 (96.7–99.7)	97.9 (96.4–99.9)	0.701	97.5 (61.7–100.2)	98.0 (93.8–102.9)	0.032
ITV_Neck_ D_98%_ [%] (*n* = 7)	98.7 (97.4–100.0)	98.0 (96.9–99.2)	–	97.0 (72.5–99.3)	98.0 (92.6–100.7)	–
ITV_Axill_ D_98%_ [%] (*n* = 3)	98.3 (98.2–98.3)	97.2 (96.8–98.3)	–	97.4 (92.2–99.4)	96.3 (94.9–98.5)	–

Body D_0.5cm^3^_ [%]	105.8 (102.9–115.9)	104.8 (101.4–106.3)	< 0.003	106.6 (103.2–116.4)	106.5 (103.3–113.0)	0.234
Body V_30%_ [cm^3^]	2047.5 (1021.2–4593.5)	3501.1 (1848.7–7805.1)	< 0.003	1895.0 (1023.7–4774.1)	3698.0 (1869.8–7935.0)	< 0.003
Breast L $\bar{D}$ [Gy_(RBE)_]	0.6 (0.0–10.0)	4.3 (1.1–13.1)	< 0.003	0.5 (0.0–10.5)	4.6 (0.9–14.1)	< 0.003
Breast R $\bar{D}$ [Gy_(RBE)_]	0.8 (0.0–9.7)	3.8 (1.4–9.2)	< 0.003	0.8 (0.0–10.9)	4.1 (1.0–11.3)	< 0.003
Breast_L V_5Gy (RBE)_ [%]	3.6 (0.0–56.2)	30.6 (3.1–88.8)	< 0.003	3.7 (0.0–59.1)	35.6 (1.3–98.2)	< 0.003
Breast_R V_5Gy (RBE)_ [%]	4.8 (0.0–43.8)	21.6 (6.5–67.4)	< 0.003	5.8 (0.0–48.2)	21.9 (3.6–73.6)	< 0.003
Heart $\bar{D}$ [Gy_(RBE)_]	3.1 (0.0–12.5)	4.6 (0.2–16.1)	< 0.003	3.3 (0.0–14.2)	5.0 (0.2–18.0)	< 0.003
Heart V_15Gy (RBE)_ [%]	8.8 (0.0–40.6)	10.2 (0.0–43.8)	< 0.003	9.5 (0.0–46.6)	11.7 (0.0–51.9)	< 0.003
LungTotal $\bar{D}$ [Gy_(RBE)_]	3.2 (0.7–6.8)	6.0 (2.6–10.1)	< 0.003	3.2 (0.4–7.7)	6.1 (2.3–10.0)	< 0.003
LungTotal V_5Gy (RBE)_ [%]	15.6 (4.5–43.3)	39.5 (4.5–78.5)	< 0.003	15.6 (2.6–49.2)	41.6 (15.3–77.8)	< 0.003
LungTotal V_20Gy (RBE)_ [%]	7.0 (0.0–14.1)	5.7 (0.0–11.5)	< 0.003	7.6 (0.0–16.0)	5.8 (0.0–11.9)	< 0.003

Recalculations on the vCTs showed that both treatment modalities generally preserved robust dose coverage to the ITV throughout treatment. Although, the ITV_Total_ coverage fell below the threshold (D_98%_ > 95%) on at least one vCT for 6/28 patients with PBS-PT, compared to only one patient for VMAT (Table 3, Fig. 2). Five out of these patients also had several vCTs below the threshold, showing that most vCTs with ITV coverage below the threshold arose from only a few patients (explicitly identified in Fig. 2). Furthermore, only two patients treated with PBS-PT showed pronounced deviation (ITV_Total_ D_98%_ < 90%), whereas no such large deviations were observed with VMAT (Table 3). The lowest observed ITV_Total_ D_98%_ was 78% for PBS-PT and 94.5% for VMAT, with the implications illustrated in Fig. 3b.

**Table 3:** Number of verifications CTs and patients with ITV D_98%_ below 95% and 90% of the prescribed dose, throughout treatment for each modality.

Target	Modality	#vCT with D_98%, ITV_ < 95%	#Patients with at least one vCT with D_98%, ITV_ < 95%	#vCT with D_98%, ITV_ < 90%	#Patients with at least one vCT with D_98%, ITV_ < 90%	Lowest D_98%, ITV_ [%]
ITV_Total_	PBS-PT	9/87 (10.3%)	6/28 (21.4%)	3/87 (3.4%)	2/28 (7.1%)	77.9
	VMAT	3/87 (3.4%)	1/28 (3.6%)	0/87 (0%)	0/28 (0%)	94.5
ITV_Med_	PBS-PT	6/87 (6.9%)	4/28 (14.3%)	3/87 (3.4%)	1/28 (3.6%)	75.9
	VMAT	1/87 (1.1%)	1/28 (3.6%)	0/87 (0%)	0/28 (0%)	94.6
ITV_FSC_	PBS-PT	5/48 (10.4%)	4/14 (28.6%)	1/48 (2.1%)	1/14 (7.1%)	61.7
	VMAT	2/48 (4.2%)	1/14 (7.1%)	0/48 (0%)	0/14 (0%)	93.8
ITV_Neck_	PBS-PT	7/25 (28.0%)	4/7 (57.1%)	5/25 (20%)	3/7 (42.9%)	72.5
	VMAT	4/25 (16%)	1/7 (14.3%)	0/25 (0%)	0/7 (0%)	92.6
ITV_Axill_	PBS-PT	2/11 (18.2%)	1/3 (33%)	0/11 (0%)	0/3 (0%)	92.2
	VMAT	1/11 (9.1%)	1/3 (33%)	0/11 (0%)	0/3 (0%)	94.9

**Fig. 2:**
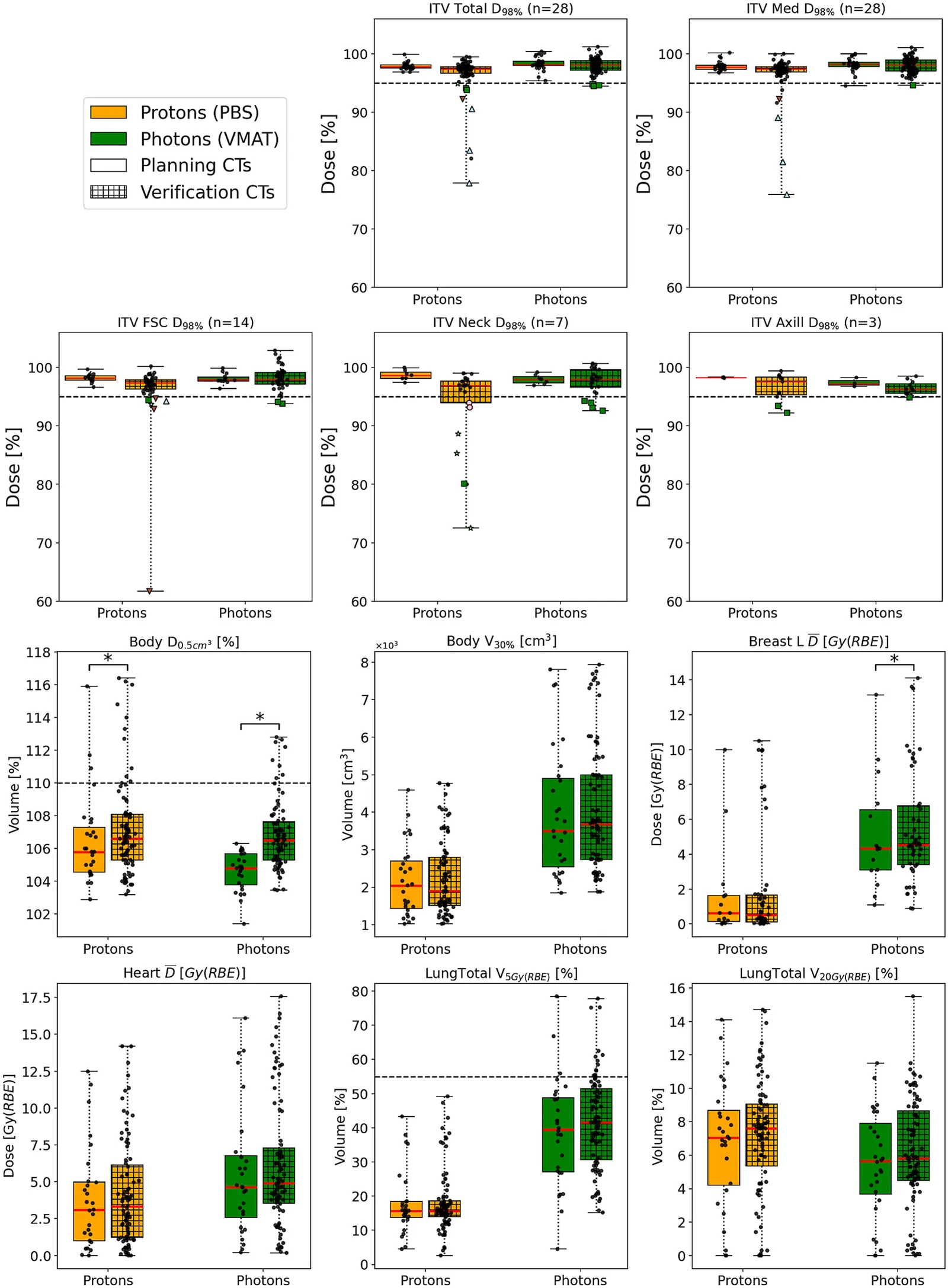
Comparison of dose-volume histogram (DVH) parameters for target structures and OOIs between planning CTs (plain, left) and verification CTs (checked, right) for proton therapy (orange) and VMAT (green). Dashed lines represent clinical criteria for the target structures and OOIs. Other OOI-metrics have established clinical criteria, for which the doses observed in this study remain far below. Some clinical OOIs criteria are simply to be as low as possible. Additionally, for each sub-target group, *n* represents the number of patients who had such a target extension. Patients with multiple vCTs below the dose target coverage threshold are indicated by unique colored symbols. * indicates significant difference (*p* < 0.003) between the planning CT and verification CT for a given modality. ITV_Neck_ and ITV_Axill_ were excluded from the statistical analysis due to small sample sizes. (For interpretation of the references to colour in this figure legend, the reader is referred to the web version of this article.)

**Fig. 3:**
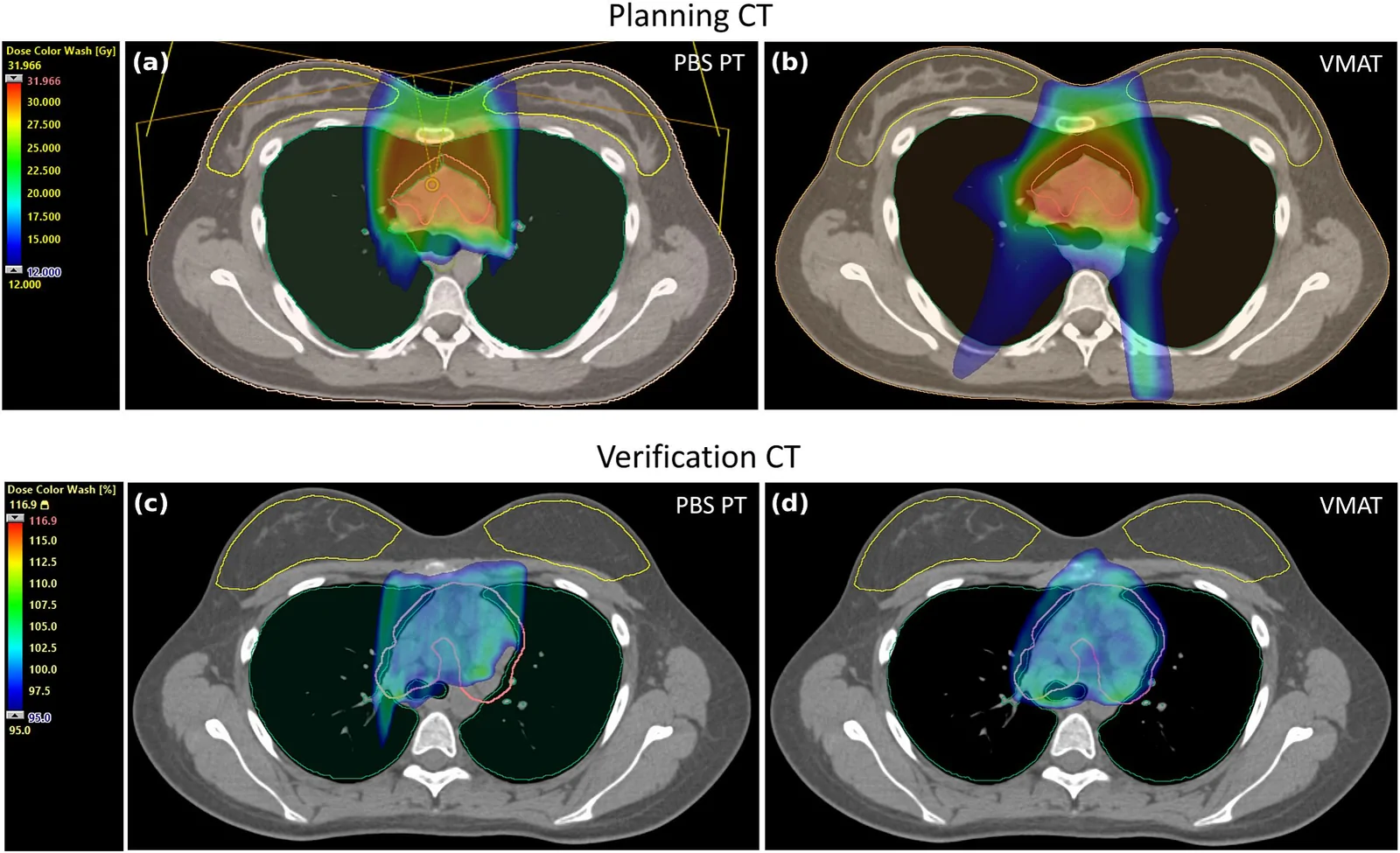
(a-b) Representative beam arrangement and dose distributions of PBS-PT (a) and VMAT (b) on the planning CT for one of the patients in the study. Cut-off-dose is 12 Gy(RBE) (34% of the prescribed dose), to demonstrate difference in the low dose-bath. (c-d) Example of recalculated dose distributions for PBS-PT (c) and VMAT (d) on the vCT with the lowest observed ITV_Total_ D_98%_ for PBS-PT. The cut-off dose is 95% of the prescribed dose to demonstrate target coverage loss. The internal target volume is shown in pink, heart in purple, lungs in green and the breasts in yellow. (For interpretation of the references to colour in this figure legend, the reader is referred to the web version of this article.)

Evaluating each sub-target independently, overall target robustness remained acceptable for both modalities (Fig. 2). However, with PBS-PT, 4/7 patients with a neck component experienced at least one vCT where ITV_Neck_ D_98%_ fell below the clinical 95% threshold, and 3/7 patients had a vCT with ITV_Neck_ D_98%_ below 90% (Table 3).

Significantly higher $\bar{D}$_L_Breast_ (0.3 Gy(RBE)) and *V*_5Gy(RBE),L_Breast_ (2.4 percentage points) were observed in the vCTs compared to pCTs for VMAT (*p* < 0.003), while both modalities showed significantly elevated D_0.5cm^3^,_ _Body_ on the vCTs, with 2.1 percentage points for VMAT and 0.5 for PBS-PT (*p* < 0.003) (Fig. 2 and Fig. S1 in the Supplementary Material).

## 4 Discussion

Both PBS-PT and VMAT plans generally maintained robust dose coverage to the ITV and OOIs throughout treatment. However, while VMAT showed greater consistency in preserving ITV coverage across vCTs during treatment, PBS-PT provided superior sparing of most OOIs. Notably, OOI doses in the nominal plans were in general significantly lower with PT compared to VMAT, with the exception of V_20Gy(RBE),LungTotal_ and D_0.5cm^3^, Body_ (significantly higher), an advantage that was preserved in the recalculated doses on the vCTs. However, more frequent instances of insufficient target coverage were seen with PBS-PT compared to VMAT, reflecting its higher sensitivity to anatomical changes and set-up uncertainties.

Despite thorough pre-treatment evaluation of the nominal PT plans and breath-hold variability, as also discussed elsewhere [18], [19], continuous monitoring of breath-hold reproducibility and anatomical changes remains essential during PT delivery. This is underscored by the observation that ITV_Total_ coverage fell below the set threshold for 6/28 patients with PBS-PT, compared with only one patient for VMAT. For this particular patient, D_98%,ITV_ fell below the clinical threshold on several vCTs with VMAT (ITV_Med_ once, ITV_Neck_ four times), and although the PBS-PT plan showed fewer instances of non-robust coverage, the deviations were larger in magnitude. This suggests that while VMAT's limitations were confined to an individual case, PBS-PT demonstrated reduced robustness across a broader patient cohort. Moreover, when suboptimal ITV coverage did occur, the impact was more pronounced with PBS-PT, as reflected by the lowest observed ITV_Total_ D_98%_ of 78% for PT versus 94.5% for VMAT (Table 3). It should be noted, however, that ITV_Total_ D_98%_ exceeded 90% for most vCTs in both PBS-PT and VMAT, with only three vCTs (two from the same patient) showing pronounced deviation for PBS-PT.

In accordance with our clinical practice, a follow-up vCT is promptly acquired if an unsatisfying dose distribution is observed in one of the weekly vCTs. The rationale is to determine whether the deviation is transient or persistent and to assess the need for replanning (described in more detail elsewhere [19]). In this patient cohort, insufficient ITV_Total_ coverage for PBS-PT triggered additional vCTs for three patients. For two of these, the second vCT again demonstrated insufficient target coverage, and replanning was performed to avoid systematic underdosage, successfully resolving the issue for the remainder of the treatment course. Although, for this study only the original plan was included in the analysis.

Dividing the total ITV into smaller sub-target volumes allowed investigation into whether particular target extensions were more affected by anatomical changes throughout treatment. Overall, target coverage on vCTs was good across all sub-targets; however, some patients exhibited noticeable ITV coverage deviations with PBS-PT (Fig. 2 and Table 3). Given the sample sizes in each subgroup, no clear correlation between target extension and reduced robustness could be established. Nevertheless, ITV_Neck_ appeared potentially more sensitive with PBS-PT, as 3/7 patients with a neck target component had at least one vCT with ITV_Neck_ D_98%_ below 90%. Additionally, in two cases, substantial D_98%_ deviations in the ITV_FSC_ and ITV_Neck_ regions (61.7% and 72.5%, respectively) were not evident when evaluating total ITV coverage alone. This suggests that in large composite targets, localized sub-volumes may experience pronounced target coverage deviations even when the overall target appears robust. These findings highlight the importance of further investigating sub-volume sensitivity to improve treatment guidelines and optimize patient selection. A potential refinement is RO on individual sub-volumes which may further improve robustness throughout treatment.

An important consideration is that all sub-targets investigated in this study were not affected directly by respiratory motion. Regions such as the neck and axilla are instead expected to be more susceptible to misalignment in arm or head/chin position. In our cohort, 5/7 patients with a neck target were treated with arms down. Although one neck-target patient treated with arms up exhibited pronounced target dose deviation (ITV_Neck,D98%_ < 90%), similar or larger deviations occurred in two patients treated with arms down. One of these arms-down patients also required replanning where the initial PBS-PT plan was complex (MFO, five fields), in which two fields covered the entire target while the remaining fields targeted individual sub-regions. Despite maintaining acceptable ITV_Total_ coverage, the plan exhibited a pronounced loss of robustness in ITV_Neck_. The other patient who required replanning (due to pronounced deviation in ITV_Med_) had a less complex SFUD plan with two fields. In that case, the primary cause of reduced robustness was inconsistent DIBH reproducibility, with substantial differences between the pCT and vCT.

Although several studies in lymphoma patients have demonstrated that nominal PBS-PT plans yield better OOI sparing than VMAT [7], [13], [21], [28], consistent with the reductions observed in this study (Table 2), these investigations have largely remained at the planning level rather than assessing robustness throughout treatment. Only few studies have assessed plan robustness during PT in DIBH. Righetto et al. evaluated 14 patients with mid-treatment CTs and found that interfractional variations remained within applied RO margins (6 mm/3.5%), with minimal impact on target coverage and OOIs [29]. Likewise, Andersson et al. reported only minor deviations in D_98%,CTV_ and OOI doses (≤0.6% and ≤ 2%) in a case report of two patients [18]. These findings support the robustness of PBS-PT, however, to our knowledge, no study has directly compared the robustness during treatment between PBS-PT and VMAT, representing a critical gap. This gap is particularly relevant, as our findings demonstrate differences between modalities, for both in the frequency (Table 3) and severity (Fig. 3) of target coverage loss due to inter-fractional variations.

During treatment, PT patients receive at least one CT scan per week for breath-hold verification and dose re-calculation, in addition to daily planar X-ray imaging for positioning. In contrast, VMAT patients are positioned with daily CBCT, which is not suitable for accurate dose calculations at our institutions. Therefore, only PT patients could be included. For PT to be selected, patients were required to demonstrate small breath-hold variability (target motion <5 mm) during the pCT session, and proton plans had to be robust to set-up and range uncertainties (7 mm/4.5%). Otherwise, VMAT was selected due to its generally greater robustness. This introduces a bias toward greater PBS-PT plan robustness, as the study cohort excluded 14 patients who did not meet PT selection criteria and instead received photon therapy. However, this bias could be further investigated in future studies, as improvements in CBCT image quality and deep learning–based dose calculation (e.g., using synthetic-CTs [30] or direct CBCT-based dose computation [31], [32]) may enable PT evaluation in the thoracic region under less stringent conditions, helping refine PBS-PT or VMAT selection.

All vCTs were acquired during a single breath-hold, assuming reproducibility throughout the fraction. Interfractional variability was partly assessed using multiple DIBH-CTs acquired at different time points; however, intrafractional and residual interfractional uncertainties remain unaccounted for. Importantly, this approach does not address the concern that prolonged treatment times may reduce breath-hold reproducibility because of patient fatigue [17]. Reduced breath-hold quality could compromise target coverage and OOI sparing, potentially to a greater extent for PBS-PT than VMAT. Therefore, minimizing treatment time by reducing the number of fields, monitor units, and required DIBHs remains important.

In conclusion, both PBS-PT and VMAT photon therapy were robust modalities for the treatment of mediastinal lymphoma in DIBH. Our findings confirmed that, in a proton-eligible DIBH cohort, PBS-PT offered a clear advantage in sparing NT and most OOIs, maintaining these benefits throughout the treatment course. However, VMAT demonstrated better consistency in maintaining target coverage, with fewer instances of underdosage observed across vCTs. This study highlights the importance of accounting for inter-fractional anatomical variations and supports the need for individualized robust evaluation, particularly when selecting PT for thoracic diseases.

## CRediT authorship contribution statement

**Filip Hörberger:** Writing – original draft, Visualization, Methodology, Investigation, Formal analysis, Conceptualization. **Marika Enmark:** Writing – review & editing, Supervision, Methodology, Formal analysis, Conceptualization. **Karin M. Andersson:** Writing – review & editing, Supervision, Methodology, Formal analysis, Conceptualization. **Rebecka Ericsson Szecsenyi:** Writing – review & editing, Investigation. **Gracinda Johansson:** Writing – review & editing, Investigation, Data curation. **Daniel Molin:** Writing – review & editing, Funding acquisition. **Kenneth Wikström:** Writing – review & editing, Methodology. **Ingrid Kristensen:** Writing – review & editing, Investigation. **Anna Flejmer:** Writing – review & editing, Methodology, Conceptualization. **Anneli Edvardsson:** Writing – review & editing, Supervision, Methodology, Formal analysis, Conceptualization.

## Ethics declaration

The authors have NOT obtained informed consent from participants or their legal representatives. This retrospective study used existing clinical data collected during routine care as part of a treatment quality improvement initiative. No additional burden, intervention, or risk was imposed on patients. The usage of radiotherapy data for retrospective research was approved by the Regional Ethical Review Board in Lund and Uppsala, Sweden (No.2013/742 and No. 2019-00010). The requirement for informed consent was waived owing to the retrospective study design.

This study was performed in compliance with relevant laws, regulatory frameworks and guidelines where the research took place.

This study was approved by the Regional Ethical Review Board in Lund and Uppsala.

(Approval No. No.2013/742 and No. 2019-00010)

## Declaration of generative AI and AI-assisted technologies in the writing process

During the preparation of this work the author(s) used ChatGPT in order to improve language and readability in selected sentences. After using this tool/service, the author(s) reviewed and edited the content as needed and take(s) full responsibility for the content of the publication.

## Funding

This research was supported by the 10.13039/501100002794Swedish Cancer Society (23 3018 Pj 01H).

## Declaration of competing interest

The authors declare that they have no known competing financial interests or personal relationships that could have appeared to influence the work reported in this paper.

## Appendix A Supplementary data

Supplementary material: Additional comparisons of dose-volume histogram parameters, similar to those shown in Fig. 2, for organs of interest between planning computed tomography (CT) images and verification CTs for both treatment modalities are available in the supplementary material.

## Footnotes

1 The neck and axilla components showed negligible variation across pCT scans, meaning CTV_Neck_ ≈ ITV_Neck_ and CTV_Axill_ ≈ ITV_Axill_; the ITV label in this study reflects the optimization on ITV_Total_, not respiratory motion of these sub-regions.
