# Supplementary material for: Comparative evaluation of plan robustness in proton and photon radiotherapy in deep inspiration breath-hold for mediastinal lymphomas throughout the treatment course
Source: Phys Imaging Radiat Oncol. 2026 Oct 22;42:101111. doi: 10.1016/j.phro.2026.101111 (PMC13742337; doi:10.1016/j.phro.2026.101111)
Supplement: Supplementary file 1 [file mmc1.pdf]

Supplementary Material: Comparative evaluation of plan robustness in proton and photon radiotherapy in deep inspiration breath-hold for mediastinal lymphomas throughout the treatment course

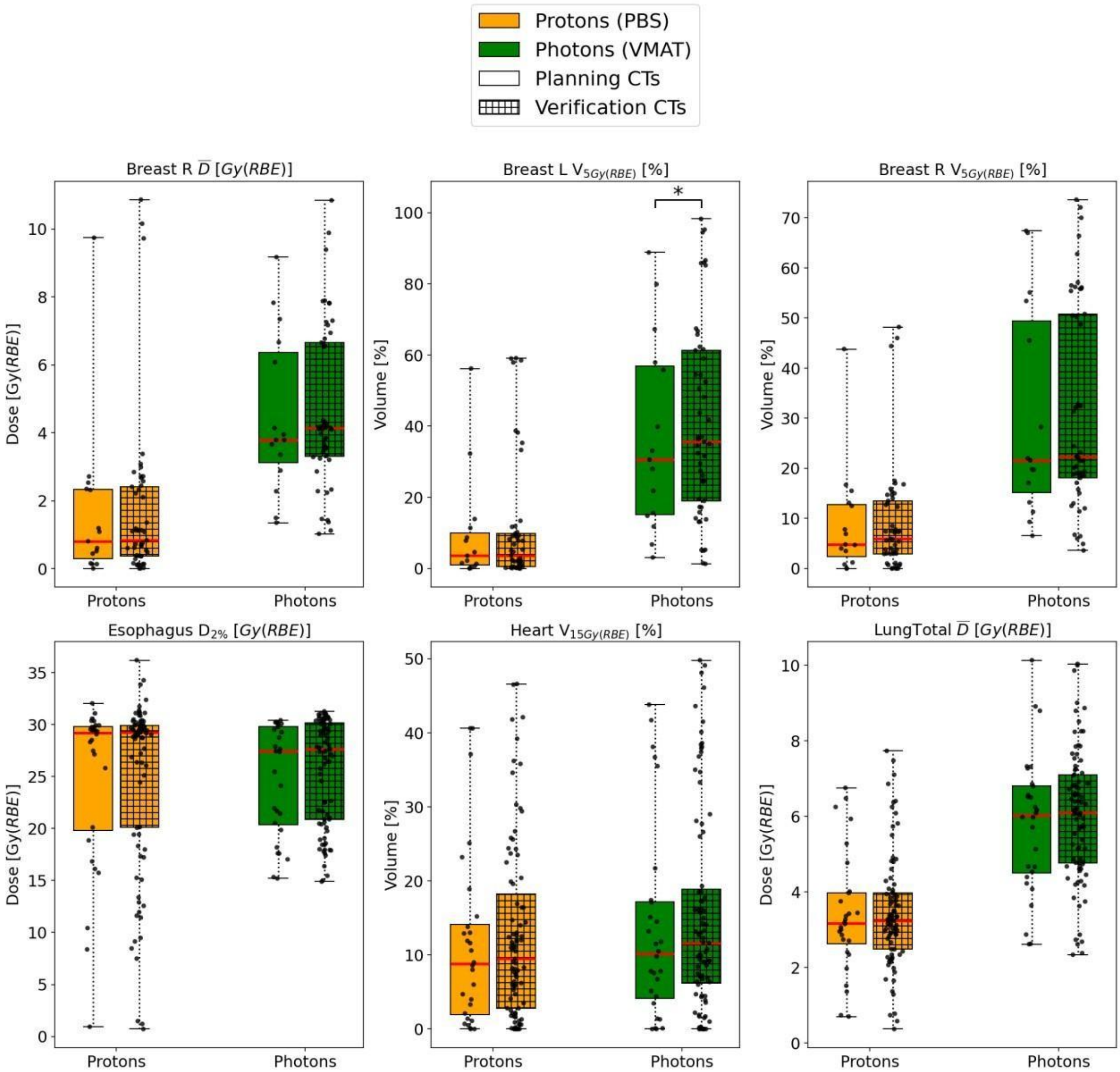

Figure S1: Comparison of dose-volume histogram (DVH) parameters for OARs between planning CTs (plain, left) and verification CTs (checked, right) for proton therapy (orange) and VMAT (green). ***** indicates significant difference ($p < 0.003$).
